# Short- and long-term effects of a quality improvement collaborative on diabetes management

**DOI:** 10.1186/1748-5908-5-94

**Published:** 2010-11-28

**Authors:** Loes MT Schouten, Marlies EJL Hulscher, Jannes JE van Everdingen, Robbert Huijsman, Louis W Niessen, Richard PTM Grol

**Affiliations:** 1 Dutch Institute for Healthcare Improvement, P.O. Box 20064, 3502 LB Utrecht, The Netherlands; 2 Radboud University Nijmegen Medical Centre, 114 IQ Healthcare, P.O. Box 9101, 6500 HB Nijmegen, The Netherlands; 3 Order of Medical Specialists, P.O. Box 20057, 3502 LB Utrecht, The Netherlands; 4 Centre for policy research, Erasmus MC, University Medical Centre Rotterdam, P.O. Box 1738, 3000 DR Rotterdam, The Netherlands; 5 Johns Hopkins School of Public Health, 615 N Wolfe Street, Baltimore, MD, USA; 6 School of Medicine, Policy and Practice, R 43/2.23, University of East Anglia, NR4 7TJ, UK

## Abstract

**Introduction:**

This study examined the short- and long-term effects of a quality improvement collaborative on patient outcomes, professional performance, and structural aspects of chronic care management of type 2 diabetes in an integrated care setting.

**Methods:**

Controlled pre- and post-intervention study assessing patient outcomes (hemoglobin A1c, cholesterol, blood pressure, weight, blood lipid levels, and smoking status), professional performance (guideline adherence), and structural aspects of chronic care management from baseline up to 24 months. Analyses were based on 1,861 patients with diabetes in six intervention and nine control regions representing 37 general practices and 13 outpatient clinics.

**Results:**

Modest but significant improvement was seen in mean systolic blood pressure (decrease by 4.0 mm Hg versus 1.6 mm Hg) and mean high density lipoprotein levels (increase by 0.12 versus 0.03 points) at two-year follow up. Positive but insignificant differences were found in hemoglobin A1c (0.3%), cholesterol, and blood lipid levels. The intervention group showed significant improvement in the percentage of patients receiving advice and instruction to examine feet, and smaller reductions in the percentage of patients receiving instruction to monitor blood glucose and visiting a dietician annually. Structural aspects of self-management and decision support also improved significantly.

**Conclusions:**

At a time of heightened national attention toward diabetes care, our results demonstrate a modest benefit of participation in a multi-institutional quality improvement collaborative focusing on integrated, patient-centered care. The effects persisted for at least 12 months after the intervention was completed.

**Trial number:**

http://clinicaltrials.gov Identifier: NCT 00160017

## Introduction

Good clinical care for patients with type 2 diabetes requires increasingly complicated drug regimens, close monitoring, and ongoing self-management support [1,2]. For best results, diabetes care also requires effective bridging of primary and specialist care with providers crossing practice and organizational boundaries [3]. Cooperation between hospitals and general practices that focus on integrated, patient-centered care is vital [4,5].

Despite a wealth of evidence and clinical practice guidelines, treatment gaps in diabetes care are visible when 'best practice' encompassing chronic care management, professional performance, and patient outcomes is compared with usual care [6,7]. Diabetes is a complex multi-systemic chronic disease and is difficult to fit into a healthcare delivery system designed to deal with acute and episodic illness. Despite reports of interventions designed to improve diabetes care, we do not know which strategies are most effective [8,9]. In a recent meta-analysis, team changes and case management showed the most robust improvements, although estimates of effectiveness of other specific quality improvement strategies may be limited by difficulty in classifying complex interventions, too few studies, and publication bias [10]. We hypothesized that a multifaceted implementation approach emphasizing collaborative learning and exchange of insights and support among a set of healthcare organizations, like a quality improvement collaborative (QIC), may be helpful to improve diabetes management and reduce risks of complications. QICs bring together a group of healthcare providers from different sites who meet periodically to learn, to exchange ideas and quality methods, and to share experiences with making changes. The aim of a QIC is to close the gap between potential and actual performance by testing and implementing changes quickly across many organizations [11]. QICs are frequently used to improve care systems in multiprofessional settings [12]. The strength of QICs is apparently the relatively efficient use of experts and peers, as well as exchange of evidence and best practices, to guide multiprofessional teams in improvement. However, the widespread acceptance and use of QICs are not based on a systematic assessment of effectiveness [13]. A recent systematic review of QICs shows varying success in achieving collaborative goals; none of the included studies provided information on the sustainability of effect [14].

To improve chronic care in an integrated care setting, a national QIC was set up to encourage high-quality, integrated diabetes care in the Netherlands. This voluntary QIC was designed to bring together and support multiprofessional diabetes teams from primary care and outpatient hospital clinics in applying evidence-based clinical practice. We hypothesized that this intervention could facilitate and support multiprofessional teamwork, integration of primary and specialist care to improve care systems, professional performance (guideline adherence), and patient outcomes.

The aim of our study was to assess the impact of this multi-institutional QIC by measuring patient outcomes, professional performance measures, and structural aspects of chronic care management. Because little is known about the impact and sustainability of QICs, the results are also meant to give insight into the short- and long-term effectiveness of this QIC in diabetes care compared to usual care.

## Methods

### Design

In a controlled, pre/post study design, the study included 15 sites representing multiprofessional provider teams from outpatient hospital clinics and general practices. We examined diabetes care for 12 months before (2004), 12 months during (2005), and 12 months after (2006) the QIC intervention.

### Participants

#### Recruitment of sites

In 2004, the Dutch Institute of Healthcare Improvement sent letters inviting diabetes provider teams in outpatient clinics and general practices nationwide to participate in a QIC in 2005. Two invitational meetings informed teams about the goals and structure of the project. The provider teams were asked to participate with at least one hospital and two or three collaborating general practices in their region so that they could form a multiprofessional improvement team. The teams consisted of medical, nursing, and allied health professionals from regional outpatient clinics and general practices. Eight geographically distinct sites volunteered to participate, and each had to pay a fee of €23,750 to cover project management costs. One site dropped out at the start of the project. The provider teams of six sites volunteered for our evaluation study (one site declined to provide evaluation data).

For each intervention site, we identified control sites with a provider team delivering usual care. The potential control and intervention sites were matched for type of site (hospital, university medical center, or general practice), location (rural or urban), size (number of beds), and teaching affiliation (yes/no). Nine sites agreed to participate in our control group.

#### Recruitment of patients

All provider teams were asked to hand out questionnaires and written consent forms for three to six weeks to patients visiting the clinic or practice. Their own physicians invited the patients to participate; patients were eligible if they were adult (older than18 years), had type 2 diabetes, were not pregnant, had a life expectancy longer than one year, and could complete a questionnaire in Dutch. In each survey, patients were asked permission for their medical records to be examined in the study. When patient recruitment stagnated, we extended the inclusion period to 10 weeks and encouraged physicians to also include patients for medical record examination only.

### Sample size

Power calculations for the sites were based on changes in mean hemoglobin A1c (HbA1c; our primary outcome measure). Assuming an expected difference of 0.4 between intervention and control sites in mean HbA1c, with a standard deviation of measurement of 1.5, alpha = 0.05, beta = 0.20, and an intracluster correlation coefficient (rho) = 0.02 [15], an average of 75 patients per site, or a total of 1,125 patients, were required for the study.

### Quality improvement intervention

The intervention sites were requested to form a multidisciplinary improvement team composed of medical, nursing, and allied health professionals from the outpatient clinic and general practices in the region. During the project, four national meetings (including one kick-off meeting) were organized to inform the participating teams about the best available evidence concerning diabetes (based on national and international guidelines), best practices and the best way to implement it. To achieve improvements, the teams were directed and supported to: change professional performance and the organization of care; introduce self-management of patients; and introduce a system to register clinical parameters. However, each team was free to focus on specific quality improvement interventions depending on service-specific routines or bottlenecks. Specific team targets included: the development of local protocols and shared care agreements between professionals (n = 5); development of local protocols focusing on the prevention of severe complications (n = 4); the communication of patient information (on the disease, its complications, the necessity of strict control, and patient partnering) (n = 2); and the monitoring of clinical indicators (n = 6). Each conference included sessions that focused on specific aspects of diabetes care, *e.g*., importance of annual follow up, targets for glycemic and cardiovascular risk control and therapy according to a step-up regimen to achieve those targets, interventions to enhance self management and lifestyle modifications, patient education and cooperation, and access to consulting services from, *e.g*., endocrinologists and diabetes educators for patients not responding to treatment or those whose diabetes is difficult to manage. A systematic approach was encouraged: the teams had to choose clear and measurable improvement aims, collect data, and plan interventions to improve care. The teams were supported by a national expert team that specializes in diabetes. Collaboration and sharing between participants was explicitly encouraged. Table 1 gives a schematic overview.

**Table 1:** Components of the quality improvement collaborative intervention

**Preparation phase**	*Context and Topic selection*
	In the Netherlands, access to care is easily available and almost fully reimbursable. Although the care for people with diabetes type 2 was mainly concentrated in primary care in the last decades, people with diabetes now receive care in primary, secondary or tertiary level care settings, The integrated care strategy intends to develop a model of care that will provide an appropriate structure to deliver the full range of health, personal, and social services and initiatives to improve the organization, management, and integration or coordination of primary generalist care and secondary specialist care services for diabetes (including diabetes specialist nurses, dieticians, podiatrists, and specialist support). Guidelines on care and prevention are amply available but not fully implemented. As part of an alliance between the Dutch Institute for Healthcare Improvement and the College of Health Insurances to improve chronic care in an integrated care setting, a national quality improvement collaborative (QIC) based on the Breakthrough Series http://www.ihi.org was set up to encourage high quality in integrated diabetes care in the Netherlands. This voluntary quality improvement strategy was designed to bring together and support multiprofessional diabetes teams from primary care and outpatient hospital clinics.
	
	*Expert meeting*
	In the preparation phase, an expert meeting of 30 national diabetes experts including general practitioners, diabetologists, specialized diabetes nurses, dieticians, podiatrists, members of the Dutch Diabetes Federation, and other patient organizations was organized. The purpose was to gain insight into current diabetes care barriers and facilitators. The experts listed 12 barriers and facilitators on the patient, professional, and organizational levels.
	
	*Expert panel and change concepts*
	Following the expert meeting, an expert panel representing five national diabetes experts and two quality improvement experts was installed to facilitate and support the participating provider teams. The expert panel prepared a package of ideas (change concepts) for closing the gap between best and actual practice. The package was based on national and international diabetes guidelines, field surveys, personal experience, and the barriers and facilitators mentioned in the expert meeting.
	
**Recruitment of participants**	*Letters of invitation*
	In 2004, letters of invitation were sent to invite diabetes provider teams in outpatient hospital clinics and general practices nationwide to participate in a diabetes QIC on in 2005.
	
	*Invitational meeting*
	In addition, two invitational meetings were organized to inform teams about the goals and structure of the project. The participating teams each had to pay a fee of €23.750 Euro to cover project management costs.
	
**Start**	*Kick-off*
	Before the kick-off meeting, the participating multidisciplinary provider teams were asked to collect some baseline data and to describe the current diabetes practice to identify 'performance gaps' in their practice. In the national kick-off meeting, the teams were provided with materials and information (package of change). The kick-off session provided information about the change package and quality improvement techniques. The topics included setting aims, the use of measurement and small, incremental tests of change.
	
**Execution phase**	*Learning Sessions*
	The teams attended three learning sessions about the change package, quality improvement methods, and reporting their experiences, changes, and results for their targets.
	
	*Plan Do Study Act (PDSA) cycles*
	Between meetings, the team members recruited other providers from their respective organizations (participating hospitals and general practices) to implement selected changes and measure progress in their own organizations. They used a PDSA change testing method to plan, implement, and evaluate many small changes in quick succession (the rapid cycle improvement method). The expert panel supported the teams by means site visits, conference calls, e-mail 'listserv' discussion groups, and feedback.

### Effect parameters

Given the diversity in improvement topics of the sites, the aim of this study was to measure the possible impact of the QIC on a wide variety of patient outcomes, professional performance, or structural aspects of diabetes care. We collected information on effect parameters (based on clinical practice guidelines) [16-19] at baseline, one year and two years follow up. We extracted patient outcomes and professional performance data from medical records and patient survey, as well as data about structural aspects of chronic care management from provider surveys.

### Patient outcomes (nine effect parameters)

To determine the collaborative's impact on patient outcomes, we assessed individual patient levels of HbA1c, systolic blood pressure, diastolic blood pressure, total cholesterol, high density lipoprotein (HDL), low density lipoprotein (LDL), body mass index (BMI), and triglycerides. On-site abstractors (nurses and practice assistants), whom we recruited, obtained patient outcome data from medical records. We provided them with detailed verbal and written instructions about the rules for scoring the biomedical items in the abstraction instrument. All available values over three years were obtained and afterwards a mean per patient per year was calculated. Data abstractors were blinded to whether the region was an intervention or control site. To assess the reliability of the data extraction, we asked the on-site abstractors to perform a re-extraction on a random subsample of 10% of the medical records on each site (intraobserver reliability 97%). Smoking status was assessed by patient survey.

### Professional performance (19 effect parameters)

Our process measures representing good clinical practice were the appropriate assessment of glycemic and cardiovascular risk control.

Based on the medical record data abstraction (as already described), we determined whether at least one measurement of HbA1c, blood pressure, total cholesterol, HDL cholesterol, LDL cholesterol, triglycerides, creatinine, urine albumin, and BMI per patient per year were performed. Data about annually foot and eye examinations, consultations with dieticians and podiatrists, and counseling (advice and instruction to monitor blood sugar, healthy diet, exercise, and smoking cessation) were obtained by patient questionnaire because medical records often do not include such data [20]. We determined binary scores for the professional performance measures, *i.e*., the patient either passed or failed the indicator (yes or no annually, measurement, examination, consultation, or instruction and advice) during baseline or one and two years follow up periods. The baseline questionnaire also requested demographics such as age, gender, and duration of diabetes; and asked about the following co-morbidities: history of foot ulcers, cardiovascular disease, stroke, renal disease, and retinopathy.

### Structural aspects of chronic care management (four effect parameters)

We used four of the six components of the Assessment of Chronic Illness Care survey http://www.improvingchroniccare.org[21-23] to assess structural aspects of chronic care management at each outpatient hospital clinic and general practice. These aspects represented the focus of improvement in our QIC intervention: self-management support, decision support, delivery system design, and clinical information systems. The provider teams were asked to complete this 19-item questionnaire three times (at baseline, after measurement, and at follow up). The respondents rated the degree to which each component (*e.g*., use of evidence-based guidelines, involvement of specialists in improving primary care, use of reminders and patient treatment plans, addressing concerns of patients and families, partnerships with community organizations, and use of information system to monitor performance, quality improvement, and to identify groups of patients needing additional care) was implemented within their diabetes care system, on a scale ranging from 0 (not at all) to 11 (fully). We computed subscale scores for each section and an overall score. A section score summed the values for all section items (*e.g*., self-management support), which was divided by the number of items within that section. The overall score was derived linearly: the average scores of each section were summed, and then divided by the number of sections.

### Statistical analysis

For both patient outcomes and professional performance measures, differences between intervention and control sites were assessed with a mixed logistic model for dichotomous outcomes, and a mixed regression model for continuous outcomes. In each model, the baseline score was entered as a covariate for correcting possible baseline differences between the intervention and control group at the start of the trial. Analyses were performed separately for short term (one year follow up) and long term (two years follow up) and differences were assessed across sites. Patient clustering within clinics and practices was accounted for. All multilevel analyses were performed with the MIXED and GLIMMIX procedures in SAS (SAS version 9.1.3, SAS Institute, Cary, North Carolina). Missing outcomes were not replaced. P values less than 0.05 were considered statistically significant. In Tables 2, 3, and 4, we present unadjusted performance scores, but the calculated significance levels are based on the abovementioned multilevel analysis.

**Table 2:** Site and patient characteristics at baseline

Site and patient characteristics at baseline	Intervention Group	Control Group
**Site characteristics**		
Number of sites participating in QIC	7	0
Number of sites participating in evaluation study	6	9
Number of hospitals	5	8
Number of general practices	12	25
Number of patients	607	1254

		

**Age in years (SD)**	**66 (12.1)**	**67 (11.2)**
**Gender, percentage of men**	**54.8**	**52.2**
**Years since diagnosis (SD)**	**13.5 (10.1)**	**13.3 (9.1)**
		
**Complications (in percentages)**		
**History of: Foot ulcer**	**11.6**	**13.4**
**Cardiovascular disease**	**22.3**	**23.3**
**Stroke**	**6.5**	**8.1**
**Renal disease**	**5.9**	**5.5**
**Retinopathy**	**10.2**	**9.1**

Patient characteristics (survey n = 1,630)		

*P = 0.01

QIC = Quality improvement collaborative

**Table 3:** Outcome measures: patient outcomes

Intermediate outcome indicators	Baseline				Short term (one year follow up)				Long term (two years follow up)			
**Medical record** **(*n *= 1,861)**	**Intervention**	**(SD)**	**Control**	**(SD)**	**Intervention**	**(SD)**	**Control**	**(SD)**	**Intervention**	**(SD)**	**Control**	**(SD)**

Mean HbA1C mmol/l (SD)	7.5	(1.3)	7.5	(1.2)	7.3	(1.2)	7.4	(1.2)	7.2	(1.2)	7.2	(1.2)

Mean systolic blood pressure mm Hg (SD)	143.3	(19.2)	143.4	(17.2)	141.6	(18.1)	141.6	(16.5)	**139.3**	(17.4)	141.8*	(16.5)

Mean diastolic blood pressure mm Hg (SD)	80.4	(8.8)	80.2	(8.9)	79.3	(8.8)	78.9	(8.6)	78.5	(9.1)	78.7	(8.6)

Mean cholesterol	4.9	(0.9)	4.9	(1.1)	4.6	(0.9)	4.6	(0.9)	4.4	(0.9)	4.5	(0.9)

Mean HDL	1.3	(0.4)	1.3	(0.4)	1.3	(0.4)	1.3	(0.4)	**1.4**	(0.4)	1.3**	(0.4)

Mean LDL	2.8	(0.9)	2.9	(0.9)	2.7	(0.9)	2.6	(2.0)	2.5	(0.8)	2.6	(2.0)

Mean BMI	29.7	(5.6)	29.6	(4.9)	29.7	(5.3)	29.5	(4.9)	29.9	(5.5)	29.7	(4.9)

Mean triglycerides	1.9	(1.1)	1.9	(1.3)	1.8	(1.1)	1.8	(1.1)	1.7	(1.1)	1.8	(1.1)

Nonsmokers (in percentages)	83.5		83.3		84.5		84.9		83.7		85.7	

*p < 0.05; **p < 0.001

BMI, Body mass index; HDL = high density lipoprotein; LDL = low density lipoprotein

Patient outcome scores are presented as unadjusted.

P value is for testing the difference between intervention and control arm at baseline and one year follow up respectively baseline and two yearsfollow up using a mixed logistic model for dichotomous outcomes, and a mixed regression model for continuous outcomes adjusting for baseline scores.

**Table 4:** Process measures: professional performance

Intermediate outcome indicators (percentage of patients)	Baseline		Short term (one year follow up)		Long term (two years follow up)	
	
	Intervention	Control	Intervention	Control	Intervention	Control
**Medical record (*n *= 1,861)**						

HbA1c checked within 12 months	**82.4**	91.5*	95.7	95.4	93.7	93.2

Blood pressure checked within 12 months	**79.4**	89.7***	89.9	93.1	88.6	91.1

Cholesterol checked within 12 months	69.4	80.1	83.2	84.3	82.2	83.4

Creatinine test within 12 months	72.9	82.1	87.8	86.9	85.5	86.8

Urine test (microalbuminuria) within 12 months	37.9	49.9	45.1	56.6	45.3	61.0

Weighed within12 months	68.7	78.7	81.2	84.8	74.5	83.5

Body mass index calculated within 12 months	22.7	33.4	43.7	39.1	41.8	43.7

						

**Survey (n = 1,630)**						

Eye examination within 12 months	**85.2**	90.9*	88.3	90.8	90.1	92.5

Foot examination within 12 months	77.5	77.8	82.7	82.7	83.0	85.2

Visit to dietician (survey) within 12 months	**29.5**	23.8*	15.8	12.8	**17.8**	9.9**

Visit to podotherapist (survey) within12 months	27.7	26.8	20.6	26.8	28.0	27.3

Received advice to self-monitor blood glucuose	72.4	66.3	69.7	64.8	68.7	65.7

Received instruction to monitor blood glucose	74.2	68.2	**59.5**	52.4*	**61.7**	55.8*

Received advice to examine feet	76.4	72.1	**77.2**	68.5*	**75.2**	69.4*

Received instruction to examine feet	64.6	59.2	**65.9**	56.3*	**66.0**	59.5*

Received advice not to gain weight	88.4	89.1	70.9	71.0	68.4	67.1

Received advice for healthful diet	94.9	93.7	75.1	71.6	72.4	67.6

Received advice for regular exercise	93.6	91.1	82.9	79.6	78.6	76.5

Received advice to stop smoking	74.6	75.7	77.9	73.0	64.6	65.8

*p < 0.05; **p < 0.01; ***p < 0.001

Performance scores are presented as unadjusted.

P value is for testing the difference between intervention and control arm at baseline and one year follow up, andbaseline and two years follow up, respectively, using a mixed logistic model for dichotomous outcomes, and a mixed regression model for continuous outcomes adjusting for baseline scores.

For assessing the impact on structural aspects for chronic care management, we performed an analysis of covariance (ANCOVA) using short-term (one year follow up) and long-term (two years follow up) outcomes, and baseline measurement as a covariate in the model.

## Results

### Study sites and patients

The 15 participating sites (six intervention and nine control sites) represented multiprofessional provider teams from 13 outpatient clinics (47 internists) and 37 general practices (42 general practitioners). Most teams had five or six members, including an internist, one or more general practitioners, a diabetes nurse, and sometimes a dietician or physiotherapist. Four intervention sites and six control sites had training affiliations. One intervention site and two control sites had university hospital links.

Table 2 shows the study sites and patient characteristics at baseline. Altogether, we collected information from medical records for 1,861 patients (*i.e*., 607 intervention patients and 1,254 control patients). A total of 1,630 patients completed the survey (583 intervention patients and 1,047 control patients). At one and two years follow up, 1,368 (84%) and 1,206 (74%) of these patients completed the survey, respectively. The mean number of patients per center was 124 (SD 44). Including only patients that provided both medical record and survey data in the analyses (n = 1,630) did not change the results.

The average patient was 66 years old, and 53% of the patients were men. Patients were diagnosed with diabetes 13 years previously on average. Approximately 22% of the patients had a history of cardiovascular disease. Participating and control patients did not differ significantly in socioeconomic characteristics (except ethnicity), history of complications, or outcome measures. The proportion of patients of Hindustani, Moroccan, or Surinamese origin was greater in the control group, mainly due to the extra number of hospitals in an urban region. Patient group analysis with and without these hospitals did not change the findings for other baseline processes and outcome measures.

### Patient outcomes

Table 3 depicts the performance changes of the patient outcome indicators for the two arms from baseline to the follow-up period. No short-term significant changes were seen between intervention and control group at one-year follow up. At two-year follow up, the mean systolic blood pressure decreased significantly by 4.0 mm Hg (from 143.3 mm Hg to 139.3 mm Hg) in the intervention group compared with 1.6 mm Hg (from 143.3 mm Hg to 141.8 mm Hg) in the control group. We also observed a statistically significant increase in mean HDL levels in the intervention group of 0.12 points compared to 0.03 points in the control group at follow up. Mean HbA1c levels diminished by 0.3% in both groups. Differences for HbA1c, blood lipids, and cholesterol levels between intervention and control group were small and insignificant. The intervention effectiveness (slope of improvement) did not differ between outpatient hospital clinics and general practices.

### Professional performance

Table 4 compares the scores of the participating and control provider teams for professional performance at baseline and follow up. Baseline adherence rates to annual examination for HbA1c, blood pressure, and eye examination were significantly higher at the control sites. The baseline rate to annual visit to a dietician was higher at the intervention group.

Some significant changes were seen between intervention and control group at one and two years follow up. The intervention group showed a modest but significant short-term improvement in the percentage of patients receiving advice and instruction to examine feet and smaller reductions in the percentage of patients receiving instruction to monitor blood glucose. These effects persisted for at least 12 months. Less worsening was seen in the percentage of patients visiting a dietician annually at two years follow up (long term). Both intervention and control sites slightly increased the number of patients with annual eye and foot examinations. Several patient information scores (received advice about...) declined over the years in both arms.

### Structural aspects of chronic care management

Thirty-five physicians from outpatient clinics and general practices (response rate 70%) provided information about structural aspects of chronic care management. Intervention group scores for self-management support and decision support differed significantly between intervention and control groups at one respectively two years follow up. Intervention group scores improved by a single point (1.0) at both one year and two years follow up; control group scores diminished by 0.5 point (Table 5). Scores for delivery system design and clinical information systems remained unchanged during the measurement periods.

**Table 5:** Structural aspects of chronic care management

Systems of care n = 35 clinics and practices	Baseline				Short term (one year follow up)				Long term (two years follow up)			
**Assessment of Chronic Illness Care (survey)**	**Intervention**	**(SD)**	**Control**	**(SD)**	**Intervention**	**(SD)**	**Control**	**(SD)**	**Intervention**	**(SD)**	**Control**	**(SD)**

Self-management support	6.0	(2.1)	6.9	(2.3)	**7.0**	(2.3)	6.4*	(2.2)	6.4	(2.2)	6.2	(2.2)

Decision support	6.8	(2.1)	7.2	(2.1)	7.7	(2.2)	6.7	(1.9)	**7.1**	(1.6)	6.4**	(2.0)

Delivery system design	7.1	(2.3)	7.8	(1.9)	7.5	(2.1)	7.8	(1.7)	7.4	(1.2)	8.0	(1.7)

Clinical information systems	6.6	(2.6)	6.4	(2.1)	6.4	(1.9)	6.1	(1.8)	7.0	(1.8)	6.7	(2.1)

												

Total mean	6.7	(2.1)	7.2	(1.8)	7.2	(1.9)	6.8	(1.7)	7.0	(1.4)	6.8	(1.8)

Total median	7.0	(2.5)	7.3	(2.1)	7.7	(2.5)	7.0	(1.9)	7.3	(1.6)	7.1	(2.1)

*p = 0.026 ; **p = 0.049

## Discussion

Our study showed modest but significant long-term effects in mean systolic blood pressure, HDL levels, scores for decision support, and less worsening in the percentage of patients visiting a dietician annually. Short term, the percentage of patients receiving advice and instruction to examine feet and scores for self-management improved significantly and less worsening was seen in the percentage of patients receiving instruction to monitor blood glucose. These changes persisted for at least 12 months after the intervention completed. We also found encouraging results on cardiovascular disease risk control at two years follow up (*i.e*. significant improvement of systolic blood pressure and HDL levels at two years follow up) [24]. The declining patient information scores (received advice about...) are intriguing. Perhaps some patient information is not provided repeatedly every year. Although we cannot exclude that patients may not remember having received an update, implicating some recall bias.

The baseline figures collected in our study are in line with the national figures in other studies [15] and our findings are consistent with the outcomes of a systematic review of QICs [14]. The results also reflect findings from other uncontrolled QIC intervention studies [25,26] and intervention studies in diabetes care [27] that show that most improvement projects produce small to modest improvement. The 0.3% difference in mean HbA1c levels in both arms is compatible with a recent meta-regression analysis [10].

Examining within group comparisons (difference in differences across intervention and control sites), the mean change (Δ) in proportion of patients with HbA1c, blood pressure, cholesterol, creatinine, and BMI checked annually increased significantly over the years at the intervention sites compared to the control sites (10 to 21% versus 1.5 to 6%; data not shown). Although some key effect parameters clearly improved in the intervention group, the observed difference between intervention and control sites (correcting for baseline scores) was modest. There are several possible reasons. First, significant baseline differences did exist for adherence rates for annual examination of HbA1c, blood pressure, eye examinations, and visits to a dietician. Given the baseline differences, both groups ultimately performed to an equivalent degree. This suggests that especially low-scoring sites were engaged to participate in the QIC, using the intervention to improve their quality of care. It also may suggest that lower baseline status facilitated greater improvement in professional performance at participating sites. Second, for some effect measures, the quality of care might be reasonably good, leading to a ceiling effect at the clinics, with little room for improvement. Third, changing diabetes management may be complex and improving patient care, particularly in a QIC context for 12 months, may generate insufficient robustness to overcome difficult organizational bottlenecks or routines [12]. Perhaps the critical mass of data and sites needed to cultivate useful exchanges of ideas, experience, and learning in the QIC was not reached [13,28]. Fourth, the specific team or organizational characteristics of the sites may have influenced the effectiveness of the QIC [12,13,28]. Although all the intervention sites improved to a certain degree, the specific interventions and the results among sites were heterogeneous. This is no surprise, given the many factors contributing to successful improvement and the likelihood that commitment to improvements, motivations, and mechanisms vary among sites [11,28,29]. We assessed key characteristics of teamwork (specific aims, type of changes initiated, degree of participation in our QIC, time, resources, composition, and climate) and organization (size, learning affiliation, and culture), but our study lacks the statistical power to justify a site-by-site analysis and only facilitates an evaluation of the QIC as a general implementation strategy. The influence of these variables therefore remained unclear. Fifth, during the collaborative period, diabetes became a national priority high on the public agenda and received much attention in professional and public media. Although the control sites remained uninvolved in organized quality improvement activities, some individual physicians or provider teams may have implemented small changes independently. Sixth, although we include a wide range of measures and based our measures on internationally accepted indicators of diabetes care, it might be possible that improvements were made, outside the scope of these measurements (*e.g*. knowledge, skills, teamwork, collaboration). In addition, QIC may have produced changes in care systems that were not large enough to significantly alter clinical processes or outcomes assessed during the evaluation period. Finally, our quasi-experimental study design has some limitations. Although we assessed the outcomes in a before/after design with concurrent controls, this was not a randomized trial. The participating sites volunteered to improve their care, not to be in a trial, so we could not randomize them to participation. Instead, we purposely selected control sites that were comparable to the participating sites. Although some significant baseline differences did exist, study sites characteristics, patient characteristics, structural aspects of diabetes care and patient outcomes did not differ significantly at baseline.

Despite these limitations, this study represents the first controlled evaluation of the collaborative methodology in the Netherlands. Even though the observed difference was modest, considering that a QIC may only be cost effective if the results are maintained, our findings on cardiovascular disease risk control and sustained effects of professional performance measures at two years follow up are promising. A concomitant paper gives an extended description of the cost effectiveness results of this trial [30].

Future research should identify collaborative, organizational and team factors associated with successful improvement to help individual teams and organizations increase the magnitude and pace of improvement.

### Summary

Healthcare systems in the US, Canada, Australia, UK, and northern European countries have adopted various types of QICs. However, few rigorously controlled evaluations have demonstrated QIC effectiveness on outcomes and sustained effect. We conclude that our QIC to improve diabetes care in an integrated care setting was associated with modest but statistically significant long-term improvements in some patient outcomes and significant improvement of aspects of professional performance and chronic care management that were sustained for at least 12 months. This suggests that gains made in a QIC can be maintained for at least a year without additional support or coaching.

## Competing interests

The authors declare that they have no competing interests.

## Authors' contributions

Authorship credit is based on substantial contribution to the concept and design, or analysis and interpretation of data, drafting the article or revising it critically for important intellectual content, and final approval of the version to be published. LS and MH obtained funding for the study, contributed to the design of study, data analysis and interpretation, and writing of paper. RG, JvE, LN and RH contributed to the design of study, data analysis and interpretation and writing the results section. All authors acknowledge that they have approved the final version of the paper submitted.
